# Sorghum Genome Sequencing by Methylation Filtration

**DOI:** 10.1371/journal.pbio.0030013

**Published:** 2005-01-04

**Authors:** Joseph A Bedell, Muhammad A Budiman, Andrew Nunberg, Robert W Citek, Dan Robbins, Joshua Jones, Elizabeth Flick, Theresa Rohlfing, Jason Fries, Kourtney Bradford, Jennifer McMenamy, Michael Smith, Heather Holeman, Bruce A Roe, Graham Wiley, Ian F Korf, Pablo D Rabinowicz, Nathan Lakey, W. Richard McCombie, Jeffrey A Jeddeloh, Robert A Martienssen

**Affiliations:** **1**Bioinformatics, Orion GenomicsSaint Louis, MissouriUnited States of America; **2**Library Construction, Orion GenomicsSaint Louis, MissouriUnited States of America; **3**Sequencing, Orion GenomicsSaint Louis, MissouriUnited States of America; **4**Biomarkers, Orion GenomicsSaint Louis, MissouriUnited States of America; **5**Department of Chemistry and Biochemistry, University of OklahomaNorman, OklahomaUnited States of America; **6**Genome Center, University of CaliforniaDavis, CaliforniaUnited States of America; **7**The Institute for Genomic Research, RockvilleMarylandUnited States of America; **8**Business, Orion GenomicsSaint Louis, MissouriUnited States of America; **9**Cold Spring Harbor Laboratory, Cold Spring HarborNew YorkUnited States of America; University of WisconsinUnited States of America

## Abstract

Sorghum bicolor is a close relative of maize and is a staple crop in Africa and much of the developing world because of its superior tolerance of arid growth conditions. We have generated sequence from the hypomethylated portion of the sorghum genome by applying methylation filtration (MF) technology. The evidence suggests that 96% of the genes have been sequence tagged, with an average coverage of 65% across their length. Remarkably, this level of gene discovery was accomplished after generating a raw coverage of less than 300 megabases of the 735-megabase genome. MF preferentially captures exons and introns, promoters, microRNAs, and simple sequence repeats, and minimizes interspersed repeats, thus providing a robust view of the functional parts of the genome. The sorghum MF sequence set is beneficial to research on sorghum and is also a powerful resource for comparative genomics among the grasses and across the entire plant kingdom. Thousands of hypothetical gene predictions in rice and *Arabidopsis* are supported by the sorghum dataset, and genomic similarities highlight evolutionarily conserved regions that will lead to a better understanding of rice and *Arabidopsis*.

## Abstract 2

Methylation filtration makes practical the sequencing of large genomes, such as those found in sorghum, by preferentially capturing functionally relevant sequences

## Introduction

Sorghum bicolor is a vitally important crop in Africa and much of the developing world. It has a remarkable ability to endure both drought conditions and water-logging and it grows well on marginal lands [1]. It is the dietary staple of more than 500 million people in more than 30 countries with only rice, wheat, maize, and potatoes feeding more people than sorghum [1]. Sorghum is in the panicoid grass subfamily and is closely related to maize, millet, and especially sugarcane, and is more distantly related to wheat and rice. Its value as a dietary staple to much of the world and its placement within the grass family make it a valuable target for genome sequencing.

Genome sequencing in most plants is difficult because of the size and complexity of the genomes. Plant genomes range in size from 54 megabases (Mb) for Cardamine amara to 124,000 Mb for a lily (*Fritillaria assyriaca*) [2]. Although they vary drastically in size, the larger genomes do not correspond to proportionally more genes, but instead to repetitive elements that have blossomed in the plant kingdom [3,4,5,6]. The extremely large genomes of such economically important crops as bread wheat (16,900 Mb), maize (2,600 Mb), soybean (1,100 Mb), and sorghum (735 Mb) [2] make them difficult to tackle with standard methods of genome sequencing such as clone-by-clone [7] and whole-genome shotgun [8]. For example, a whole-genome shotgun project of maize to 8× genome equivalents would require nearly 24 million sequencing reads, and sorghum would require 7.5 million reads. Additionally, the maize and sorghum genomes are more than 75% repetitive [9,10], which would make the final assembly of shotgun sequence extremely difficult [11]. The large-insert clone-by-clone approach solves some of the difficult assembly problems, but it requires a much larger initial investment in resources and is much more expensive. Furthermore, the highly repetitive large-insert clones would still be difficult to assemble.

Evidence has accumulated over the last ten years that many plant genomes are separated into large tracts of methylated repeats and stretches of hypomethylated, low-copy gene–rich space [4,6,12,13,14,15]. On the basis of this knowledge of plant genome architecture, two techniques have been developed to isolate the low-copy or hypomethylated regions of the genome for sequencing. The first technology, high C_0_ *t* selection (C_0_ *t* is the product of the DNA concentration [C_0_] and the reassociation time in seconds [*t*]), allows the separation of low-copy sequences from those of high copy based on annealing rates [16,17]. High C_0_ *t* selection has been used successfully to sequence the low-copy, genic regions of maize [18] and has been applied to sorghum [10]. The second technology is methylation filtration (MF), which preferentially clones the hypomethylated fraction of the genome. MF has also been successfully applied in maize to sequence the genic regions [18,19,20]. It appears that MF will be a successful strategy across the plant kingdom, as it has been shown to enrich for genes in all plants tested, from monocots to dicots to gymnosperms, and even in nonvascular plants such as moss (P.D. Rabinowicz, unpublished data).

We have applied MF technology to generate sequence from the hypomethylated portion of the sorghum genome. Successful sequencing of fewer than 550,000 MF reads revealed that approximately 96% of the gene set of sorghum has been sequence-tagged, with an average coverage of 65% across their length. Because MF targets genomic sequence within and around genes, many important components of the genome are represented, including promoters, microRNAs (miRNAs), introns, simple sequence repeats (SSRs), and potentially active transposable elements.

The sorghum gene space is a powerful resource for comparative genomics within the grass family and across the plant kingdom. The MF dataset can be used to confirm hypothetical genes in complete genomes such as rice and *Arabidopsis* and to identify functional elements conserved across different plant species.

## Results/Discussion

### The Size of the Genome Space Sampled by MF

To calculate the genome space sampled by MF, two independent methods were used, genome sampling and gene-enrichment. Genome sampling is an empirical calculation based on a modification of the Lander-Waterman equations [21], as used by Whitelaw and colleagues [18]. The reduced genome size is calculated based on the size of the sampled space as judged by the number of times that independent reads overlap. Independent reads will overlap more often when sampling a small region versus a larger region; therefore, one can derive an empirical assessment of the size of the region being sampled [18]. The sampled genome space for the sorghum MF set is 262 Mb.

The gene enrichment method works on the assumption that genes are enriched in the MF libraries in proportion to the reduction in genome size. For example, if the genome is reduced by 3-fold, then gene discovery should occur 3-fold faster in MF versus whole-genome shotgun libraries. The extent to which this number agrees with the genome sampling method is the extent to which the genes reside in the sampled space. We calculate gene enrichment because it can be estimated very early in a sequencing project, whereas the genome sampling method requires at least 0.1× coverage of the sampled space to get an accurate estimate (unpublished data).

The gene enrichment factor is called filter power (FP); we use FP to derive the sampled genome space by dividing it into the size of the whole genome. We calculated the sorghum FP using a subset of our filtered and unfiltered (UF) sequences compared to a curated database of known genes over a range of BLAST Expect values (E-values) (Table 1). The FP is between 3.0 and 3.8 with a median value of 3.15. By dividing this range of FP values into the 735 Mb sorghum genome, the sampled genome is estimated to be between 193 Mb and 245 Mb, with a median of 233 Mb. The median estimate is somewhat lower than the 262 Mb estimation derived by the genome sampling method. However, the result depends critically on genome size estimates, which for S. bicolor range from 735 Mb to 858 Mb [2]. If 858 Mb is used, gene enrichment predicts a 272-Mb gene space, which is slightly higher than the 262 Mb obtained by genome sampling, thus bracketing the genome sampling approximation, depending not only on the range of FP, but on the range of genome size estimates.

**Table 1:**
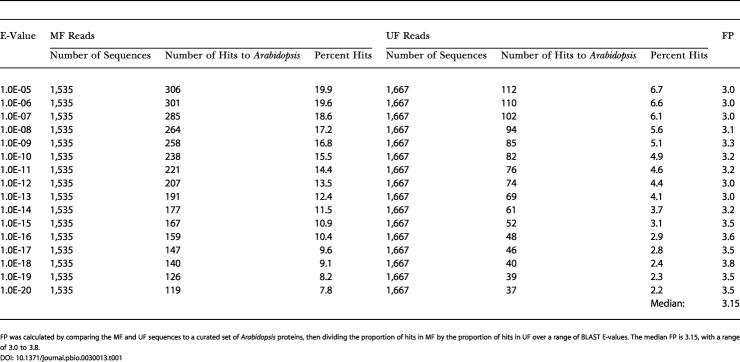
Gene Enrichment (or FP) of MF Versus UF Sequences

FP was calculated by comparing the MF and UF sequences to a curated set of *Arabidopsis* proteins, then dividing the proportion of hits in MF by the proportion of hits in UF over a range of BLAST E-values. The median FP is 3.15, with a range of 3.0 to 3.8

**Figure 1:**
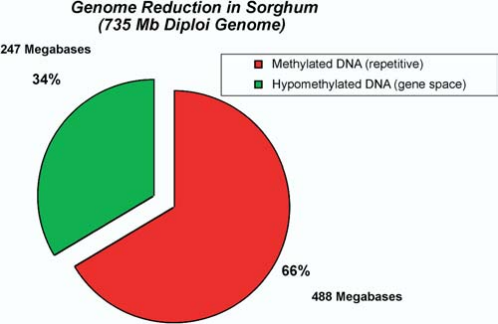
Genome Reduction MF reduces the sorghum genome by 66% in sampling a hypomethylated space of approximately 247 Mb (green) and filtering out 488 Mb (red) of the 735-Mb sorghum genome.

Therefore, completely independent estimates of gene space, namely genome sampling and gene enrichment, agree well and are within measurement error. For the purposes of this manuscript, 247 Mb, which is the average of the two methods, will be used as an approximation of the sampled, hypomethylated genome space (Figure 1). The MF dataset consists of a nuclear coverage, after collapsing read pairs, of 285 Mb, which is approximately 1.15× coverage of the sampled space.

### Gene Tagging and Coverage

**Figure 2:**
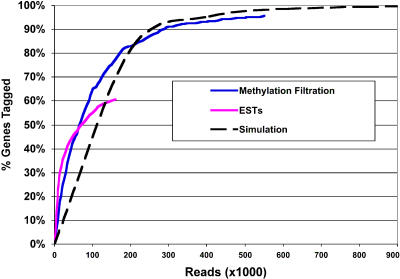
Gene Discovery Rate Gene discovery rates for sorghum MF (blue), sorghum ESTs (pink), and an *Arabidopsis* simulation (dotted black) are shown. The gene discovery rates for the MF and ESTs were calculated based on matches to a set of 137 genes annotated on sorghum BAC clones versus the number of MF and EST reads. The *Arabidopsis* simulation was calculated based on the fold-coverage of chromosome 1, which contains 7,520 genes. The fold coverage was converted into read numbers as detailed in the Materials and Methods.

The purpose of a genome reduction method such as MF is to identify genes in a robust and efficient manner. We assessed the efficiency of gene discovery by calculating the percentage of known genes tagged as a function of read number for MF and compared this value to the rate of gene discovery obtained by expressed sequence tags (ESTs) for sorghum (Figure 2). Additionally, we conducted a simulation in *Arabidopsis* to assess the expected gene identification rate in a completed plant genome where the level of coverage could be controlled precisely in silico (see Expected Gene Tagging, below). The results of these analyses are summarized in Figure 2.

To estimate the percentage of genes that have been tagged by MF, we used high-quality sorghum bacterial artificial chromosome (BAC) sequences as a source of gene annotations. At the time of analysis, 14 finished sorghum BACs had been deposited in GenBank (http://www.ncbi.nlm.nih.gov/). Because the GenBank annotations were outdated, we reannotated the BACs through a custom annotation pipeline (see Materials and Methods). We annotated a total of 148 genes on these BACs, then mapped our MF reads to the BACs using stringent BLAST criteria. Of the 148 genes, the MF reads match 133 (90%) of them, with an average nucleotide coverage of 61%.

However, 11 of the 148 annotations are alpha kafirin storage protein genes on BAC AF527808. Ten of them constitute a tandem repeat cluster of nearly identical sequences that could be expected to be methylated [22] and are therefore not recovered efficiently in a MF library. This is indeed the case, as only two out of the 11, or 18%, are recovered in the MF clones. This is far below the 90% average for the whole set, suggesting that the kafirin genes may be at least partially methylated (see Methylated Gene Recovery, below). If we remove these 11 genes from the analysis, 131 (95.6% [Figure 2]) of the remaining 137 genes are tagged across 65% of their nucleotides. We also removed the kafirin genes from the EST analysis in Figure 2.

In addition to tagging 95.6% of the gene set, a majority of the coding sequence (CDS), upstream, and downstream genomic regions are covered. The average coverage of the CDS regions of all 137 genes is 65%, thus providing a tag across more than half of the gene on average. This coverage is consistent with the 67% nucleotide coverage predicted at 1.15× raw sequence coverage [21]. Additionally, we calculated the nucleotide coverage 500 basepairs (bp) upstream (5′) and downstream (3′) of the CDS and found 74% and 69% coverage, respectively. The coverage of the 5′ and 3′ regions is higher than expected, which is at least partly due to the close spacing of sorghum genes in this set, with 16/137 (greater than 10%) having 5′ and/or 3′ regions within 1 kb.

For comparison, the gene tagging ability of the publicly available sorghum EST sequences was assessed. At the time of analysis, there were 161,766 sorghum ESTs deposited in GenBank. Using criteria of 98% identity over at least 50 bp of the CDS, the sorghum ESTs matched 84/137 (61%) of the annotated BAC genes (Figure 2). Notably, the ESTs did not match any of the 11 kafirin genes.

### Expected Gene Tagging: An *Arabidopsis* Simulation

If MF faithfully represents the genic region of sorghum and contains the vast majority of the genes, then the rate of gene tagging should produce results that are similar to whole-genome shotgun coverage [21] at the same level of raw coverage. To test this hypothesis, we simulated a whole-genome shotgun project of the finished *Arabidopsis* chromosome 1 (see Materials and Methods). We decided to use *Arabidopsis* for the simulation because it is finished to high quality, the gene predictions are the most robust of any plant species, and *Arabidopsis* best represents the size of plant genes, which are much smaller on average than animal genes.

The simulation showed that, at 1.1× coverage, 96.4% of the genes are sequence-tagged across 66.8% of their length. These numbers are very similar to the percentages calculated from the MF gene tagging analysis (95.6% of genes covered over 65%). Since the simulation is set to replicate Lander-Waterman whole-genome shotgun conditions, these results mean that MF obeys the mathematics of Lander-Waterman, although it is a highly fragmented sampling space. Furthermore, if the BAC gene set is representative of the genome, this implies that nearly all the genes in the genome are accessible to MF and that all genes are currently covered over an average of 65% of their length. Theoretically, 100% nucleotide coverage will be reached at 6× coverage, which would require less than 2.5 million additional MF reads.

Figure 2 shows the comparison of the gene tagging rates for the *Arabidopsis* simulation, the MF reads, and the sorghum ESTs. Notice that the gene tagging for the sorghum ESTs and MF are more rapid than the *Arabidopsis* simulation. Rather than reflecting a real difference in ability to tag genes using MF versus whole-genome shotgun, this higher rate likely reflects the larger average gene length for the sorghum CDS annotations (3 kb) versus *Arabidopsis* (2.3 kb), making gene tagging more rapid in sorghum. Additionally, the sorghum ESTs show the most rapid gene-tagging rate, but begin to level off at 60% gene tagging and are passed by the sorghum MF after 70,000 reads.

### Methylation of Transposons, Repeats, and Pseudogenes

Overall, recognizable repeats constitute 62% of the sorghum genome (Table 2, Unfiltered), which is comparable to maize [18,19]. Retrotransposons are the most abundant class of repetitive DNA sequence, occupying about 1/3 of the genome, followed distantly by DNA transposons at 1/20 of the genome (Table 2, Unfiltered). MF reduces the recovery of ribosomal genes, centromeric repeats, and retrotransposable elements (Table 2, Filtered), so that only 27% of filtered reads match repeats. These results can be described in terms of the total fraction of repeats (*R/N*, where *R* is the total length of repeats in the genome and *N* is the size of the genome), the unmethylated fraction of repeats (*r/UM*, where *r* is total length of repeatsin the unmethylated fraction and *UM* is the size of the unmethylated genome), and the filter power (FP) (*N/UM*) according to Palmer and others [19]. Given a FP of 3.15 (*N/UM*), we can calculate the proportion of unmethylated repeats (*r/R*) as (*[r/UM]/[R/N]*)/(*N/UM*), or approximately 10%. This is consistent with maize [19], and indicates that a substantial portion of sorghum transposons, especially DNA transposons, are unmethylated and may be capable of transposition. For example, the active sorghum transposon *Candystripe1*(*Cs1*) [23] is represented in our dataset across 23% of its length (unpublished data). The lower-than-average percent coverage (23% versus 66%) may be due to some methylation within the element, as has been reported for several maize transposons [12]. Additionally, *Cs1* is known to have a low copy number (less than 10) in sorghum, and the redundancy of coverage across the 23% represented suggests that MF is sampling from a single element (unpublished data).

**Table 2:**
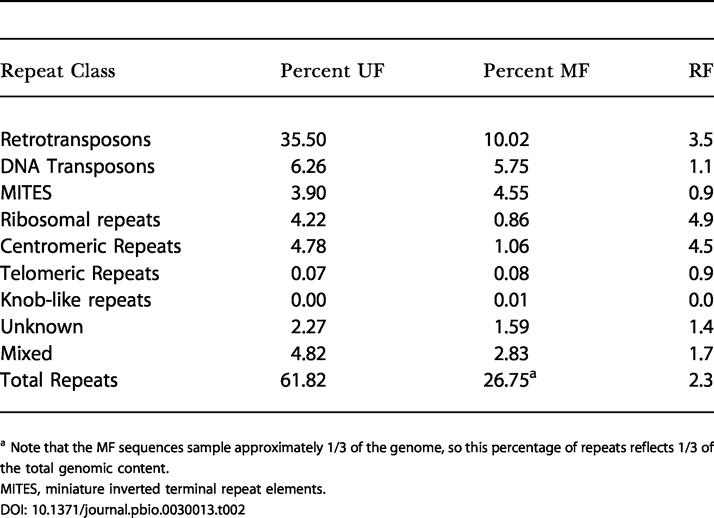
Repeat Analysis for MF Versus UF Reads

^a^ Note that the MF sequences sample approximately 1/3 of the genome, so this percentage of repeats reflects 1/3 of the total genomic content

MITES, miniature inverted terminal repeat elements

**Figure 3:**
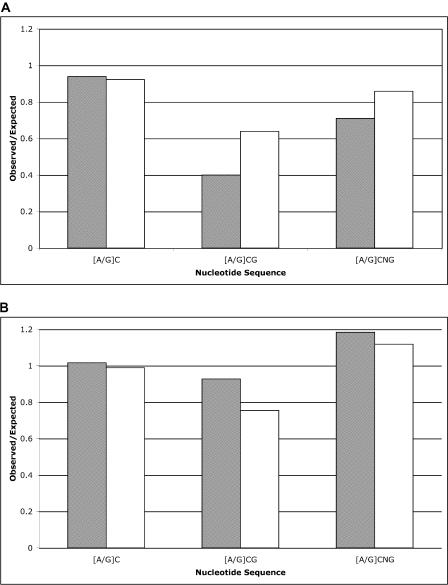
CG and CNG Suppression in MF versus UF Sequences Sequences were analyzed for their mcrBC half-sites, those that overlap CG dinucleotides, and those that overlap CNG trinucleotides. The ratio of observed to expected sites is graphed for filtered (hatched) and unfiltered (white) for retrotransposons (A) and CDSs (B).

The majority of methylation in plants occurs at the canonical sites CG and CNG (where N is any nucleotide) [24,25,26,27]. MF uses in vivo restriction via modified cytosine restriction, subunits BC (mcrBC) at the recognition site (A/G) methylated cytosine (mC). The observed versus expected occurrences of mcrBC sites, along with those sites that overlap the canonical methylation sites of CG and CNG, are shown for retrotransposons and genic sequences in Figure 3A and 3B, respectively. Although the mcrBC half-sites ([A/G] C) occur as expected in MF and UF retrotransposons and genes, the sites that overlap canonical methylation sites are significantly reduced in MF versus UF retrotransposons, but not in genic sequence, where, in fact, they occur more frequently in MF than UF (Figure 3). It has been shown previously that CG and CNG nucleotides are suppressed in MF repetitive elements [19], presumably because mCs have been converted over time to thymine by deamination [28]. Our results suggest that such conversion has occurred in transposon sequences, but not in genes, consistent with their differential methylation.

The increased frequency of CG and CNG nucleotides in genic sequences recovered by MF versus UF (Figure 3B) suggests that CDS derived from MF and UF are different. One source of this difference may be the presence of pseudogenes. In plants, most pseudogenes are marked by small insertions and deletions, resulting in frame shift(s) of the coding region, but are otherwise indistinguishable from functional genes [29]. Pseudogenes are likely targets of silencing and are thus probably methylated, excluding them from MF sequences. To test if pseudogenes are more abundant in the UF dataset, sequences from both UF and MF that matched *Arabidopsis* proteins, and are therefore considered genes, were compared to a database of all plant proteins using BLASTX. Sequences with more than one high-scoring segment pair and with an E-value of 1 × 10^−20^ or less were analyzed for the presence of a frame shift. The rate of potential frame shifts for UF is 103/530 (19.4%) versus 1,599/17,103 (9.35%) for MF, indicating that pseudogenes are recovered at a higher rate in UF (comparable to the rate of retrotransposons) and are therefore most likely methylated.

### Methylated Gene Recovery

**Figure 4:**
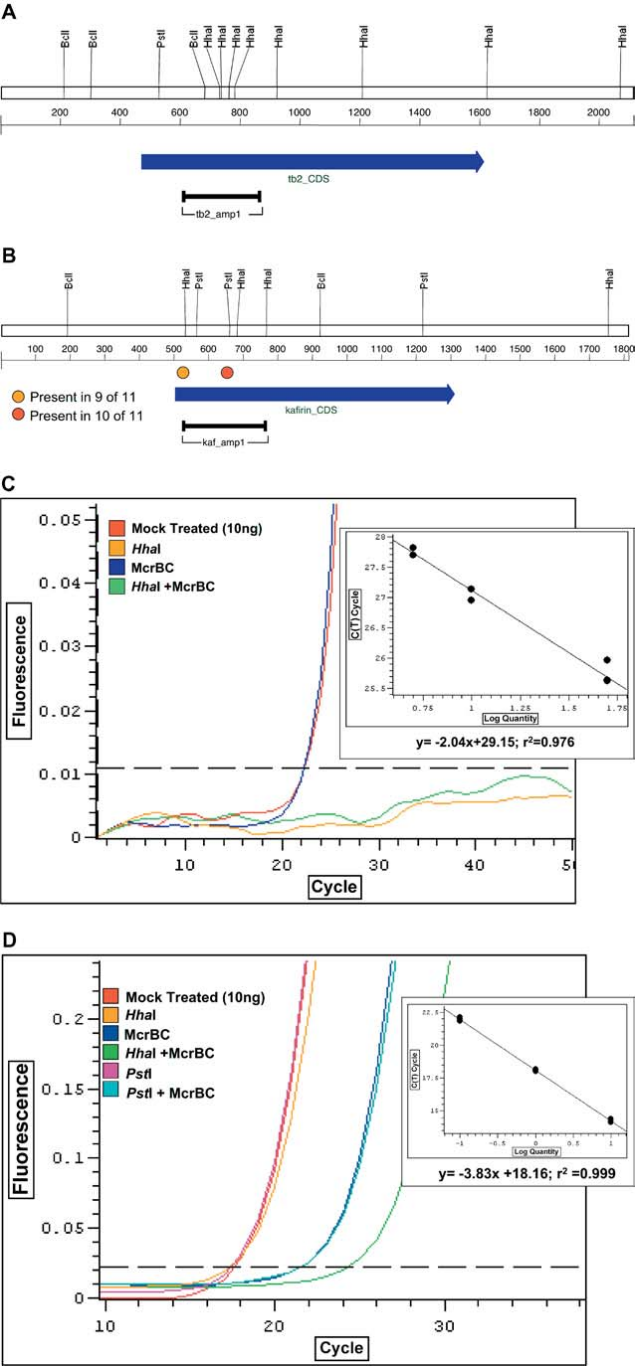
Methylation Status of *tb2* and Kafirin Cluster (A and B) Restriction maps of the *tb2* gene (A) and the kafirin consensus sequences (B) are shown. The relevant restriction sites are indicated vertically and the numbers indicate the distances scale in basepairs. Each CDS is depicted as a blue-shaded arrow, and the region assayed is indicated by a black bar. The circles depict sites that are not present in every kafirin gene, and the color represents the number of genes that do not share the site. The orange circle (5′-most HhaI site) is a site conserved in nine of 11 kafirin genes, and the red circle (3′-most PstI site) is a site present in ten of the 11. (C) Results from a representative methylation analysis of *tb2;* the inset depicts the template dilution standard curve used to set the threshold for the experiment. Each experiment was performed three times with four on-board replicates per assay point. The results for each of the four differentially treated reactions are depicted with different colors. Red, mock-treated; blue, mcrBC-digested; orange, HhaI-digested; and green, HhaI + mcrBC double-digest. The inset shows the standard dilution control with two replicates at each dilution. The control was used to set the threshold for detection. The specificity of each reaction was confirmed using melt-curve analysis. (D) Results from a representative methylation analysis of the 11 kafirin genes. The results for each of the six differentially treated reactions are the same as in (C), with the following additional digests: pink, PstI-digested; light blue, PstI + mcrBC double-digest. Notice that the mcrBC with and without PstI yields the same Ct, while HhaI + mcrBC (green) yields a higher Ct on average; suggesting additional cleavage.

Comparison with the BAC sequences revealed that a small number of genes were not represented in the sorghum MF reads. Two explanations were considered: First, these genes may have been missed by chance, as only 97% of sorghum genes were expected to be sampled by this depth of coverage. Second, these genes might be methylated. Two examples were chosen for further analysis: the *teosinte branched2* gene (*tb2*), which was recovered in our dataset, and the kafirin storage protein gene cluster (Figure 4). The kafirin gene cluster was chosen because it is underrepresented in the MF sequences and could be methylated since it is a tandem repeat cluster [22]. We used a real-time PCR technology to assess DNA methylation (see Materials and Methods). As expected, methylation analysis of *tb2* (on BAC AF466204) indicates that it is unmethylated (Figure 4A and 4C).

For the kafirin gene cluster, only two of 11 genes from BAC clone AF527808 were represented in the MF dataset, suggesting that most or all of them may be methylated. Ten of the genes are tandemly arrayed in a cluster and share an average of 99.1% sequence identity, while the eleventh gene is located 45 kb away and is more diverged (76.2% identity on average). A 247-bp region was selected for PCR close to the 5′ end because of its near identity across all 11 genes and because of the high CG and CNG content (Figure 4B). The methylation results are depicted in Figure 4D. PstI sites are methylated (at CNG), since the PstI-treated sample (Figure 4D, pink) has the same cycle threshold (Ct) as the mock-treated sample (Figure 4D, red). This result is supported by the mcrBC digested sample, which has a significantly higher Ct (Figure 4D, dark blue) than the mock-treated DNA control. All, or almost all, of the PstI sites are methylated, because the double PstI +McrBC digest (Figure 4D, light blue) has the same Ct as mcrBC alone (Figure 4D, dark blue). These results indicate that every gene has CNG methylation covering these sites.

As for CG methylation, the HhaI-digested (orange) sample has the same Ct as the mock-treated control (red); however, the Ct of the HhaI + McrBC double digest (green) is 2.46 cycles greater than the mcrBC alone (dark blue), indicating that some HhaI sites must not be modified. A cycle threshold difference of 2.46 indicates that there is 2^2.46^, or approximately 5.5-fold, less DNA in the HhaI + mcrBC double-digested sample. This suggests that two out of the 11 kafirin genes have some unmethylated HhaI sites.

To determine which kafirin genes might be unmethylated, we sequenced the kafirin PCR products from mcrBC treated and untreated genomic DNA (gDNA). 130 sequences from mcrBC-treated DNA and 126 sequences from the mock-treated sample were analyzed. The kafirin genes fall into “subfamilies” based on six polymorphisms within this highly conserved genomic region (see Materials and Methods). Each of these subfamilies was represented among the sequenced clones, including the orphaned kafirin gene outside the tandem array, indicating that none was completely removed as a consequence of mcrBC treatment. Thus, it is likely that all the kafirin genes contain some level of methylation, and that the genes are displaying nonuniform CG methylation randomly, perhaps on a per-cell basis, across all 11 genes.

### Drought Resistance Genes

In order to assess how useful the current low level (approximately 1×) coverage of the gene space is for answering important comparative genomics questions, we chose to analyze genes related to drought resistance. Sorghum's ability to grow in arid conditions makes it an attractive source of genes to enhance drought resistance in other grasses. Part of the drought-responsive pathway in plants involves the activation of dehydration-responsive element binding protein (DREB) transcription factors belonging to the APETALA2 (AP2) family. The overexpression of DREB1-encoding genes can promote drought, freezing, and salinity tolerance in transgenic plants [30].

**Figure 5:**
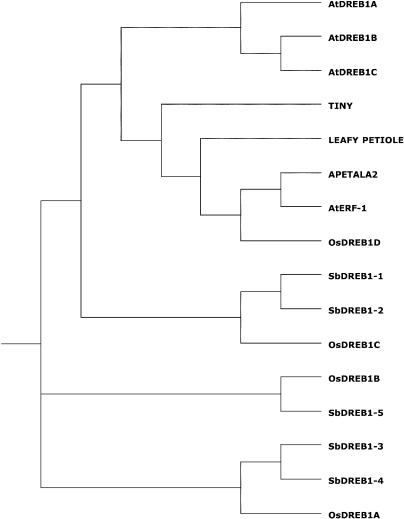
Phylogenetic Comparison of Sorghum *DREB1* Genes A phylogenetic tree comparing the AP2 domain of the sorghum *DREB1* genes to those of *Arabidopsis* and rice was constructed using CLUSTALX [61]. The genes encoding proteins from *Arabidopsis* are *DREB1A, DREB1B,* and *DREB1C*. Rice genes are *OsDREB1A, OsDREB1B, OsDREB1C* (nucleotides 142,337–142,981), and *OsDREB1D* (nucleotides 1,489–2,250). AP2 domains from other *Arabidopsis* proteins are also included: *APETALA2* (R2 domain), *AtERF-1, LEAFY PETIOLE,* and *TINY*.

A screen of the sorghum MF dataset reveals five full-length DREB1-like proteins, based on conservation of the AP2 domain and a conserved C-terminal LWSY motif (see Materials and Methods). A phylogenetic tree constructed from the AP2 domains of the *Arabidopsis*, rice, and sorghum DREB1-encoding genes suggests that sorghum has expanded the *DREB1* family and that *SbDREB1–1* and *SbDREB1–2* are the closest orthologs to the *Arabidopsis* DREB1 family (Figure 5). This analysis also suggests that the rice gene *OsDREB1D* may not belong to the *DREB1* family, a hypothesis supported by the fact that *OsDREB1D* does not contain the conserved LWSY motif and its expression was not detected under drought, freezing, or salt-stress conditions [31]. An expansion of the *DREB1* family in sorghum may contribute to the plant's enhanced drought resistance. Certainly the identification of other sorghum genes involved in the drought response regulatory pathway is now possible. This analysis highlights the utility of this dataset in answering fundamental comparative biology questions even at such a low level of gene space coverage.

### Global Comparisons to Rice and *Arabidopsis*

In order to assess the utility of the sorghum MF set for cross-genome annotations, we compared the annotation of rice by sorghum MF versus the complete gene set in *Arabidopsis*. The rice genes were downloaded from The Institute for Genomic Research (TIGR) and contain the genomic sequence of gene predictions, which includes exons and introns. The rice set contains 57,535 genes that we categorized into known (23,115), hypothetical (21,438), and repetitive (12,982), based on the annotation (see Materials and Methods).

The rice sequence was used as the query in searches of sorghum MF and *Arabidopsis* proteins. A rice gene was considered supported if it had a best match better than or equal to a BLAST E-value of 1 × 10^−8^. Of the rice gene set, 46,450 (81%) had a match to sorghum MF, while only 38,462 (67%) matched *Arabidopsis*. The matches can be further broken down by category, with 22,282 (96%) of known rice genes, 13,262 (62%) of hypothetical rice genes, and 10,906 (84%) rice repeats matched by sorghum MF. In comparison, *Arabidopsis* annotated 20,827 (90%) known, 7,850 (37%) hypothetical, and 9,785 (75%) repeats. Thus, the 1.15× coverage of the closely related sorghum gene space does a much better job of providing supporting evidence for gene predictions in rice than does *Arabidopsis*. Interestingly, the number of hypothetical genes matched by sorghum MF is almost 2-fold higher than that annotated by *Arabidopsis*. This may indicate a higher proportion of grass-specific genes in the hypothetical predictions.

To understand how well cross-species gene annotation is accomplished in a low-redundancy MF versus a nearly complete genome, we compared the annotation of *Arabidopsis* by sorghum MF to that by rice. Such a comparison provides a good test of annotation capacity without being complicated by different evolutionary distances, since *Arabidopsis,* being dicotyledonous, is expected to be the same evolutionary distance from both sorghum and rice.

**Figure 6:**
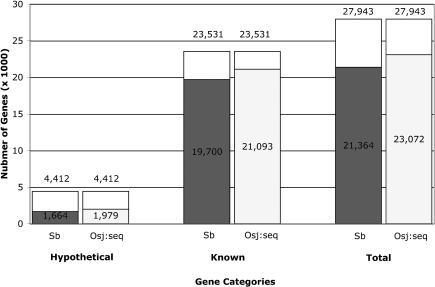
Annotation of *Arabidopsis* by Sorghum MF Versus Rice Gene Sequences Shown are the number of *Arabidopsis* proteins that are matched in a TBLASTN comparison to the sorghum MF set (blue) versus the rice gene sequences (yellow). The *Arabidopsis* proteins, after having known repetitive elements removed (see Materials and Methods), have been categorized as either hypothetical or known based on the definition line. *Arabidopsis* proteins were considered supported if they matched with an E-value less than or equal to 1 × 10^−8^. Sb, S. bicolor MF set; Osj:seq, *Oryza sativa japonica* gene sequences.

An *Arabidopsis* protein was considered supported if it had a BLAST match less than or equal to an E-value of 1 × 10^−8^ (Figure 6). In this analysis, 19,700 (84%) of the known and 1,664 (38%) of the hypothetical proteins had a match to sorghum MF, whereas 21,093 (90%) of the known and 1,979 (45%) of the hypothetical proteins had a match to rice. This indicates, as expected, that a complete monocot genome is a better tool for annotating a dicot than is a partial genome; however, the difference is not that big, suggesting that a low level, cost-effective skim of many different genomes for comparative genomics may be more economical than complete sequencing.

Interestingly, although the rice sequences match more *Arabidopsis* proteins than sorghum, the set is not completely overlapping, and sorghum matches 247 proteins that are not matched by the rice sequences. Since we used rice gene predictions as our database for comparison, it is likely that some of the *Arabidopsis* proteins are in the genome but are not annotated as genes. To address this possibility, we compared the 247 *Arabidopsis* proteins to the entire rice genome (*Oryza sativa japonica*) and found that 59 did indeed match to the bare gDNA versus the annotations, and therefore were not unique to the sorghum-*Arabidopsis* genomes. That left 188 proteins that may be conserved in sorghum and *Arabidopsis,* but not in rice. The *O. s. japonica* genome was sequenced by the BAC-by-BAC method [32], and it is likely that some regions are not represented in the BAC clones. Therefore, we compared these 188 to the *O. s. indica* genome, which was sequenced by whole-genome shotgun [33] and would have different biases than BAC-by-BAC. Again, a proportion (61) of these were found in the genome under our BLAST criteria, leaving 127 *Arabidopsis* proteins that are supported by sorghum but either missing from or significantly diverged in the current versions of the *O. s. japonica* and *O. s. indica* genomes (Table S1). Laboratory experiments will be needed to confirm that these are truly missing from rice; if they are missing, they represent an interesting set of genes that could highlight previously unknown shared features between sorghum and *Arabidopsis* to the exclusion of rice. For example, the myb-related protein CAPRICE, a gene involved in root-hair cell development [34,35], was in this set, which may indicate a previously unknown conserved root development pathway in sorghum and *Arabidopsis* to the exclusion of rice.

### MiRNAs

MiRNAs are a class of small RNAs that are important in gene regulation through recognition and cleavage of target mRNA. They are short sequences, usually 18–24 nucleotides in length, that match target genes and gene families, although usually imperfectly. Regulation is achieved through cleavage by the RNAi silencing complex. They are encoded by hairpin precursors that are processed in at least two steps by RNase III-domain ribonucleases related to *Dicer*. MiRNAs have been found in all eukaryotes surveyed and seem to be well conserved between plant species [36,37,38].

We downloaded 122 and 92 known rice and *Arabidopsis* miRNAs, respectively [39], and used them in a BLAST search against the sorghum MF set. Of these, 91 (75%) of the rice miRNAs and 44 (48%) of the *Arabidopsis* miRNAs had exact matches in the sorghum MF set (Table 3). For comparison, the miRNAs were searched against the completed rice genome, sorghum ESTs, and maize MF + HC (high C_0_ *t*) assemblies, with 121, 16, and 88 of the rice miRNAs and 52, 10, and 46 of the *Arabidopsis* miRNAs matching, respectively.

**Table 3:**
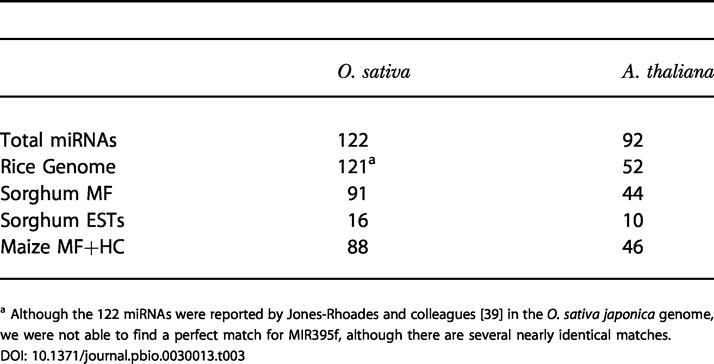
MiRNA Content in Sorghum, Rice, and Maize

^a^ Although the 122 miRNAs were reported by Jones-Rhoades and colleagues [39] in the *O. sativa japonica* genome, we were not able to find a perfect match for MIR395f, although there are several nearly identical matches

**Figure 7:**
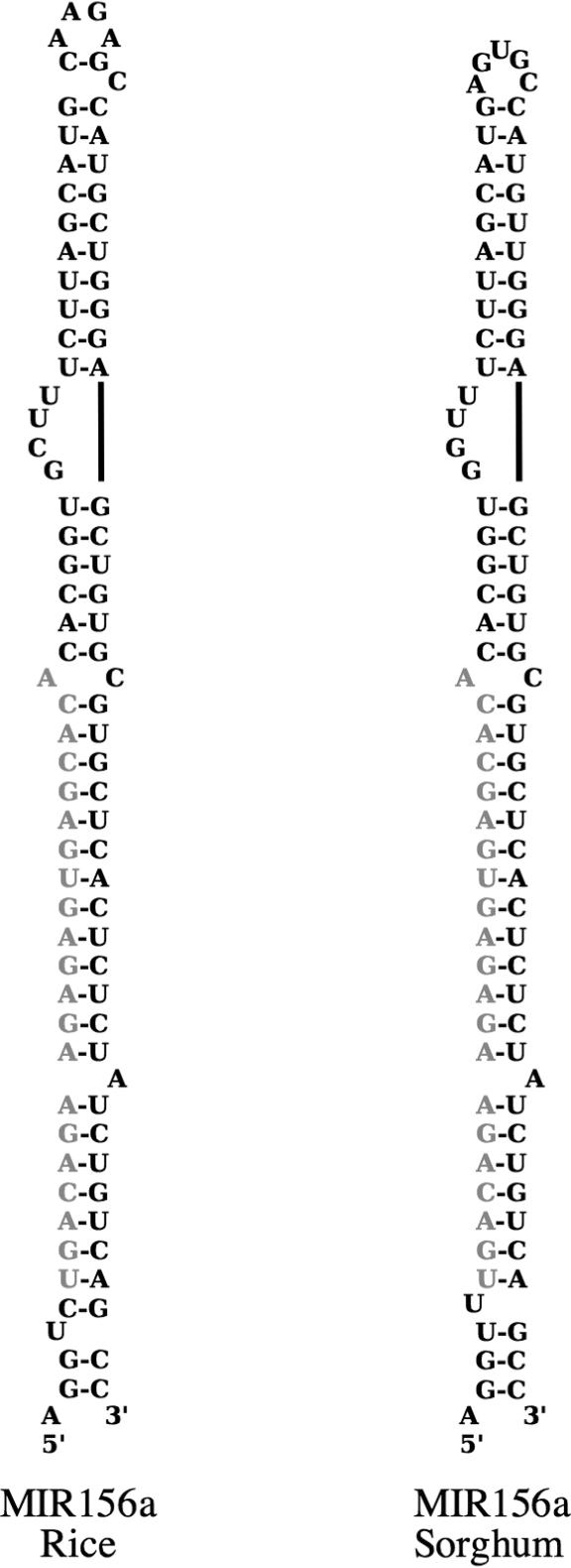
Secondary Structure of Predicted MiRNAs Predicted hairpin secondary structure of miRNA MIR156a from rice and the newly discovered ortholog from sorghum. The 21-nucleotide MIR156a sequence is highlighted in red.

To ensure that these were authentic matches and not just due to chance, we performed a test with shuffled miRNA sequences, maintaining the nucleotide composition (see Materials and Methods). None of the shuffled sequences matched any of the databases, indicating that the matches are authentic and not due to the small size or a biased nucleotide composition of the miRNAs. Additionally, precursor sequences surrounding these miRNAs could form hairpins (Figure 7 and unpublished data), and were also matched by rice gDNA, indicating they are likely to encode the corresponding miRNA.

We do not know a priori how many of the rice miRNAs would be expected to be conserved in sorghum, but we can assume that most, if not all, of the miRNAs conserved between *Arabidopsis* and rice would also be conserved between *Arabidopsis* and sorghum. Therefore, given that the rice genome is nearly complete, we expect to find the same 52 *Arabidopsis* miRNAs in sorghum, and we have identified 44 (85%). The eight that are missing may be present in the data but not identified because of sequencing errors; not yet sampled, as we expect only approximately 66% of the nucleotides to be present at this level of coverage; or some of these eight may represent miRNAs conserved in rice but lost in sorghum.

### Simple Sequence Repeats

SSRs are stretches of DNA with simple sequence pattern repetitions, usually in the form of di-, tri-, or tetra-nucleotide expansions such as (CA)n, (CAG)n, or ( GATA)n. These stretches of DNA are useful for genetic marker analysis, because they are unstable and often are polymorphic between closely related individuals [40,41]. Overall, SSRs are enriched in MF sorghum sequences, 22,445 of 417,113 (5.4%), compared to UF, 335 of 17,276 (1.9%), indicating that most SSRs are unmethylated. GC-rich trinucleotide repeat (TNR) SSRs in plants have been shown to be preferentially associated with coding regions [42,43]. We observe an increase in the proportion of GC-rich TNRs to total TNRs in MF sequences, 6,464 of 8,957 (72%), compared with whole-genome shotgun, 63 of 129 (49%). This observation suggests that this collection of sorghum sequences is laden with new and publicly available molecular breeding and genetic mapping tools.

### The Sorghum Genome and Comparative Genomics

The sequence of the sorghum gene space provides an excellent tool for comparative genomics [44]. Unlike maize, which it otherwise resembles, sorghum has not undergone recent genome duplications, although there is evidence for ancient duplications in most cereal genomes [45]. For this reason, sorghum and rice share a greater degree of colinearity than maize and rice [40], potentially facilitating mapping of quantitative traits across these three genomes, including drought resistance [46]. Sorghum is also a close relative of sugarcane (*Saccharum* spp.), whose large and variable chromosome content makes genome sequencing impractical. The availability of a large number of sugarcane EST sequences [47] will enable comparison of these genomes to identify genes of potential agronomic value in this species as well. Such comparisons will extend even to the large collection of microsatellite SSR markers reported [41]. The sequence reported here is an important first step for these comparisons.

The sorghum gene set present in the MF data is very nearly complete, as illustrated by the ability to annotate *Arabidopsis* nearly as well as the completed rice genome and by the ability to identify 95% of the genes from finished sorghum BACs. This was achieved with a minimal sequencing effort, which brings within reach the prospect of sequencing multiple strains of the same species. Such a feat is of critical importance in maize, in which inbred lines differ substantially in gene order and content [48,49].

### Sequencing Large Plant Genomes Using MF

A disadvantage of gene enrichment strategies, whether they are EST sequencing, high C_0_ *t* selection, or MF, is that the recovered fragments are not positioned on the genome. Mapping has to be accomplished by either mapping the reads to a physical or genetic map or by combining the gene enrichment with an anchored clone map. MF reads are enriched for SSRs, which make good genetic markers and allow some reads to be placed on a genetic map. If a framework physical map of fingerprinted BAC clones exists, then MF can be easily integrated onto the physical map in three ways: PCR mapping to BAC pools, hybridization to BAC filters, and/or by sequence integration. Sequence integration can be accomplished using either BAC end sequence or shotgun sequence from a representative tiling path of the BAC contigs. While there is no whole genome BAC map of sorghum yet available, a robust map is almost complete in maize [50,51]. It is estimated that a BAC tile of maize will consist of approximately 18,000 BAC clones. Skim-sequencing from these clones at approximately 1× coverage, combined with a deep coverage through gene enrichment, are predicted to generate a high-quality sequence map for a fraction of the cost of whole genome sequencing [48]. BAC sequencing projects are ongoing for sorghum [40], which can use the MF reads in much the same way to enhance the BAC shotgun sequence and speed the completion of the genome.

## Materials and Methods

### 

#### MF library construction

Seeds of S. bicolor ATX623, kindly provided by J. Osborne (NC+ Hybrids, Colwich, Kansas, United States), were germinated and grown in soil under growth chamber conditions. Then gDNA was purified from isolated nuclei of 1-mo-old leaves as described [52], except that OptiPrep (Axis-Shield PoC, Oslo, Norway) was used. Shearing of nuclear DNA was performed using either a nebulizer (Cis-Us, Bedford, Massachusetts, United States) or Hydroshear (GeneMachines, San Carlos, California, United States). Sheared fragments were end-repaired using a variety of enzymes including mungbean nuclease, T4 DNA polymerase, Klenow fragment, and T4 polynucleotide kinase. End-repaired fragments were size-selected on an agarose gel and DNA fragments ranging from 0.7 to 1.5 kb were extracted and ligated to dephosphorylated, HincII-digested pBC SK– vector (Stratagene, La Jolla, California, United States) which was used to construct both MF (GeneThresher technology; Orion Genomics, Saint Louis, Missori, United States) and UF libraries. Ligation reactions were transformed into mcrBC+ and mcrBC– strains of E. coli for generation of MF and UF libraries respectively. Recombinant clones were picked using Genetix Q-bot robot (Research Genetics, Carlsbad, California, United States) and stored individually in 384-well microtiter plates.

#### Sequence data

Two sources of MF sequencing reads were used. Out of 604,641 attempts at Orion Genomics, 532,150 were successful (accession numbers CL147592–CL197752 and CW020594–CW502582), 514,983 of which are considered of nuclear origin based on comparison with chloroplast, mitochondrial, viral, and bacterial databases. Additionally, we have included 36,825 sorghum MF reads previously generated at Cold Spring Harbor Laboratories (Cold Spring Harbor, New York, United States) (accession numbers CC058553–CC059980, BZ329127–BZ342789, BZ342901–BZ352342, BZ365856–BZ368372, BZ369686–BZ370012, BZ421595–BZ424357, BZ625682–BZ629992, and BZ779555–BZ781928). The sorghum UF sequences also came from two sources: Orion Genomics (1,819 reads) (accession numbers CW512190–CW514008) and the University of Oklahoma (15,889 reads) (NCBI TraceDB accession numbers TI566112507–TI566128395). The average read lengths were 600 bp and 550 bp for each class of reads, giving a total, raw nuclear dataset of approximately 330 Mb (MF) and 10.5 Mb (UF). The MF dataset was further collapsed by assembling overlapping read pairs to generate a set of independent sampling events comprising approximately 285 Mb.

#### Database curation and FP calculation

We have done a first pass definition-line curation of publicly available sequence databases to eliminate obvious transposon sequences that would hamper subsequent analyses by virtue of inflating the true “gene” content of the given database.

The *Arabidopsis* protein set, which was used for the gene enrichment calculations and assessment of cross-genome annotation potential, was downloaded from the NCBI (ftp://ftp.ncbi.nih.gov/genomes/Arabidopsis_thaliana/CHR_*/*.faa). The files were dated 23 May 2003 and contained 28,581 sequences (12,112,846 total letters). Repeats were removed from this dataset if the definition line meets both of the following two criteria: (1) Matched the case-insensitive regular expression “/retro|mutator|transpos|reverse transcriptase|polyprotein|\bgag\b|BARE-1|athila/”, and (2) did not match “/\[.*retro.*\]|leucine|WD-repeat|WD repeat|WD40|WD-40| ankyrin|telomere|arm repeat|PPR-repeat|armadillo|tetratricopeptide|TPR-repeat|TPR repeat|Kelch|pentapeptide|C-repeat/”.

This second step was used to replace falsely identified nonrepetitive elements. Removing repeats reduced the database size by 640 sequences to 27,941, which included 4,412 sequences identified as hypothetical by matching the definition line to the case-insensitive regular expression “/hypothetical/.”

The rice sequence set was downloaded from TIGR (ftp://ftp.tigr.org/pub/data/Eukaryotic_Projects/o_sativa/annotation_dbs/pseudomolecules/version_2.0/all_chrs/all.seq). The file was dated 30 April 2004 and contained 57,535 sequences (155,419,428 total bases). The rice sequence set contains the genomic regions for all predicted rice genes, which includes exons, introns, and untranslated regions where good evidence is provided. No sequences were removed from this database, but they were classified as “repeats,” “hypothetical,” and “known” by the following criteria. (1) Sequences were classified as repeats if the definition line matched a case-insensitive regular expression “/retro|transpos|reverse transcriptase|gag|polyprotein|mutator|maggy|rire|gypsy|copia|bare-1/”; (2) the sequences were classified as hypothetical if the definition line matched a case-insensitive regular expression “/hypothetical/”; and (3) the remaining sequences were classified as known. In total, there were 13,008 repeats, 21,441 hypotheticals, and 25,263 known proteins. The rice chromosomal genomic sequences were used for miRNA identification (ftp://ftp.tigr.org/pub/data/Eukaryotic_Projects/o_sativa/annotation_dbs/pseudomolecules/version_1.0/all_chrs/all.con) and dated 05 September 2003. It contains 12 chromosomes, with sequences comprising 358,546,960 bases.

Sorghum ESTs were download from the NCBI (ftp://ftp.ncbi.nih.gov/genbank/*.seq.gz and ftp://ftp.ncbi.nih.gov/genbank/daily-nc/nc*.flat.gz, which was last dated 20 October 2003, and contains 161,766 sequences (83,411,684 total bases). No sequences were removed from this database.

Gene enrichment was calculated by comparing the rate of gene discovery between MF and UF sequences. To ensure high quality, unique sampling events, reads were chosen that contained at least 100 contiguous Phred Q20 bases and only one read per clone. Detection of genes was accomplished by an NCBI-BLASTX search (parameters: -e 0.01; -b 5; -v 5) of the curated *Arabidopsis* protein database (see Materials and Methods). Aside from the curation of the *Arabidopsis* database to remove repetitive elements, matches to proteins annotated as hypothetical were not counted. Hypothetical genes are often false gene predictions or unknown repetitive elements. In order to calculate a gene enrichment factor, or FP, the proportion of matches from MF sequences are compared to the proportion of matches in UF sequences over a range of E-values from 1 × 10^−5^ to 1 × 10^−20^, such that all matches better than the given E-value are tabulated (Table 1). For sorghum, the genome size is estimated at 735 Mb [2]. Dividing the genome size by the median 3.15 FP provides an estimate of a 233 Mb sampled space.

#### BAC annotation

There were 14 finished BAC clones at the time of analysis, with the following accession numbers (and GenInfo identifiers). AC120496.1 (GI:20486389), AF010283.1 (GI:2735839), AF061282.1 (GI:4539654), AF114171.1 (GI:4680196), AF124045.1 (GI:5410347), AF369906.1 (GI:19851516), AF466199.1 (GI:18390096), AF466200.1 (GI:18481699), AF466201.1 (GI:18483227), AF466204.1 (GI:18568251), AF503433.1 (GI:21326110), AF527807.1 (GI:22208458), AF527808.1 (GI:22208471), and AF527809.1 (GI:22208503). The BACs were manually annotated, then reads were mapped to the BACs by BLAST to determine the locations of hits relative to the genes.

We analyzed the BACs with several computational tools in addition to manual editing. Repetitive elements were identified using RepeatMasker [53] with the MaskerAid speed enhancement [54] and the TIGR cereal repeat database. The TIGR cereal repeat database, dated 11 July 2003, was downloaded (ftp://ftp.tigr.org/pub/data/TIGR_Plant_Repeats/) and contained 11,043 repeat entries. RepeatMasker was run with the following parameters: “-s; -w; -no_is; -nolow; -lib cereal_repbase.lib”. RepeatMasker parameters also included “-xsmall” to mask in lowercase and “-w” to use the MaskerAid [54] enhancement. To look for known protein-coding genes, we searched each repeat-masked BAC against all plant proteins with WU-BLASTX 2.0MP-Washu (23 May 2003) [55,56] using a serial strategy [57]. The first search used the parameters W=5; V=0; E=1e-5; X=10; nogap; kap; altscore=“* any na”; altscore=“any * na”; wordmask=seg; lcmask. The second search used default parameters. To look for transcript similarities, we searched all plant transcripts with WU-BLASTN using a serial strategy with the following first-round parameters: W=12; V=0; X=7. In the second round we used the parameter: W=9. Both BLASTN searches had these additional parameters: wordmask=seg; lcmask; M=1; *N*=–1; Q=3; R=3; kap; E=1e-10; hspmax=0. To look for potentially novel genes, we used Fgenesh (http://www.softberry.com/berry.phtml) with monocot parameters, Genscan [58] with *Arabidopsis* parameters, and SNAP [59] with *Arabidopsis* parameters.

In order to annotate the locations of genes in each BAC, we loaded all the computational results into the ACEDB viewer (http://www.acedb.org) and edited gene structures by hand. One of the challenges was how to determine when the tools had identified pseudogenes. These are often marked by adjacent repeats, BLASTX alignments containing stop codons, or gene predictions with tiny introns to compensate for frame-shifts. Another challenge was how to use cross-species alignments. Alignments that are nearly identical to genomic sequence are useful for delimiting exon boundaries, but inexact matches pose problems because alignments may terminate because of real exon boundaries or differences between the sequences. Since most of the alignments were from plants other than *S. bicolor,* we did not employ any programs that align a protein or transcript directly to a genome. Instead, we assigned the position of the splice sites in part by consulting exon predictions, since gene finding algorithms contain probabilistic models of splice sites. We did not report any raw gene predictions. However, some genes do contain exons with no overlapping evidence and are included in the gene structure because they complete an otherwise incomplete gene structure and in some cases are necessary to maintain the reading frame. The BAC annotations are available in GFF format with the supplemental online data.

The sorghum MF sequences were compared to the collection of 14 sorghum BACs using NCBI-BLASTN (parameters: -p blastn; -F ‘m D'; -e 0.01; -b 14; -v 14). A sequence was considered mapped to a BAC if the match was over 90% of the read with 98% identity or higher. A single read was mapped to only one location. A gene was considered tagged if one or more MF sequence(s) overlapped the CDS region by 50 bases or more. The set of S. bicolor ESTs were mapped to the BACs using the same BLAST parameters, but a gene was considered tagged using less stringent criteria, since genomic introns will not align. A gene is tagged by an EST if it aligns at 98% identity over at least 50 bp, but there was no requirement for the percentage of the EST that needed to be aligned.

#### *Arabidopsis* simulation

A computational simulation of shotgun sequencing *Arabidopsis* chromosome 1 was compared to the empirical gene tagging results in sorghum. The sequence and annotation of *Arabidopsis* chromosome 1 was downloaded from TIGR (ftp://ftp.tigr.org/pub/data/a_thaliana/ath1) on 20 February 2004. The chromosome is approximately 30 Mb long with 7,520 genes annotated. The median gene size is 1,960 bp.

Computationally generated “reads” of 700 bp in length were created from the chromosome for different levels of raw coverage from 0.5× up to 3.5×. The reads were then mapped back to the chromosome annotation to determine the percentage of the 7,520 genes that were tagged at each level of raw coverage (results shown in Figure 2). The percent gene tagging was calculated on a fold-coverage basis (e.g., 0.5×, 1.0×, etc.), so in order to convert it to a meaningful read number basis for Figure 2, we converted the fold-coverage to a number of reads by using the estimated genome space (247 Mb) divided by the average sorghum read size (604 bp), resulting in approximately 409,000 reads per 1× coverage.

#### MiRNA analysis

The A. thaliana and O. sativa miRNAs were downloaded from the supplementary online material for Jones-Rhoades and colleagues [39]. This dataset contains 122 and 92 computationally predicted and experimentally confirmed miRNAs for Oryza sativa and Arabidopsis thaliana, respectively. The miRNAs are grouped into 18 and 22 families for rice and *Arabidopsis*, respectively. These sequences were used in a WU-BLASTN [55] search of the MF sorghum set (parameters: -W 18; -M 1; -N −4; -Q 1; -R 1; -wordmask=seg; -warnings). A match was scored if the miRNA matched at 100% identity over its complete length. The same parameters were used for the rice genome, sorghum ESTs, and maize MF + HC databases. The maize MF + HC database is release 4.0 of the Zea mays MF and HC combined assembly from TIGR (http://www.tigr.org/tdb/tgi/maize/).

In order to test the specificity of these miRNA matches, we generated shuffled sequences for the 122 rice and 92 *Arabidopsis* miRNAs. The shuffling maintains the nucleotide composition of each while scrambling the order [60]. The shuffled sequences were used in WU-BLAST searches against all the databases with the same parameters as above. None of the shuffled sequences had an identical match to any database. These results indicate that the miRNAs are not matching simply because of their small size and nucleotide composition, but probably represent authentic evolutionarily conserved units.

#### Comparison with rice and *Arabidopsis*.

The rice sequences were compared to the sorghum MF using NCBI-BLASTN with the rice sequences as the query and the sorghum MF reads as the database (parameters: -p blastn; -b 1500; -v 1500; -r 1; -q -1; -G 2; -E 1; -F ‘mD'; -e 1e-5). We counted a rice gene as hit if the E-value was less than or equal to 1 × 10^−8^, which corresponds to a bit score of approximately 61. The rice hits were then counted and categorized.

To assess how well rice is annotated by a dicot, the rice sequences were also searched against the *Arabidopsis* protein set using NCBI-BLASTX (parameters: -p blastx; -e 1e-5; -F ‘mS'). We counted a rice gene as hit if the E-value was less than or equal to 1 × 10^−8^, which corresponds to a bit score of approximately 51. The rice genes hit were counted and categorized.

The *Arabidopsis* protein set was compared the sorghum MF dataset using NCBI-TBLASTN (parameters: -p tblastn; -e 1e-5; -F ‘mS'). We counted an *Arabidopsis* protein as hit if the E-value was less than or equal to 1 × 10^−8^, which corresponded to a bit score of approximately 57. The *Arabidopsis* hits were then counted and categorized as shown in Figure 6.

The *Arabidopsis* protein set was also compared to the rice gene sequence dataset using NCBI-TBLASTN (parameters: -p tblastn; -e 1e-5; -F ‘mS'). We counted an *Arabidopsis* protein as hit if the E-value was less than or equal to 1 × 10^−8^, which corresponded to a bit score of at least 57. The *Arabidopsis* hits were then counted and categorized as shown in Figure 6.

There were 247 *Arabidopsis* proteins that were annotated by sorghum MF but not rice sequence. These 247 proteins were then compared to the entire rice genome using NCBI-TBLASTN (parameters: -p tblastn; -e 1e-5; -F ‘mS'). From that set of 247 we removed any *Arabidopsis* proteins if the E-value was less than or equal to 1 × 10^−8^, which corresponded to a bit score of at least 59. The remaining 188 proteins were then compared to the entire genome from the *O. s. indica* cultivar of rice [33] using NCBI-TBLASTN (parameters: -p tblastn; -e 1e-5; -F ‘mS'). From that set of 188 we removed any *Arabidopsis* proteins if the E-value was less than or equal to 1 × 10^−8^, which corresponded to a bit score of approximately 60. The resulting set contained 127 *Arabidopsis* proteins that were supported by sorghum MF but not found in the rice genomes.

#### Methylation analysis

Methylation was assessed using MethylScreen analysis, which is a real-time PCR technique that reports DNA methylation occupancy information for genomic markers through enzymatic interrogation. MethylScreen analysis compares the cycle thresholds (Cts) of gDNA that has been subjected to various treatments and infers 5′ methylated cytosine (5 mC) occupancy through the changes in Ct mediated by the treatments. The Ct of any locus is a function of the number of copies present within the assay tube. MethylScreen analysis relies upon the simple formula that total gene copies = number of methylated copies + number of unmethylated copies in every sample. Typical sample assays utilize four sample subportions, the first portion of a sample is mock-digested, reporting total copies present. A second (and equal) portion is treated with a methylation-sensitive restriction enzyme (MSRE), reporting the number of gene copies that are methylated. The third portion is digested with a methylation-dependent restriction enzyme (MDRE) such as mcrBC, reporting the number of copies that are unmethylated. The fourth reaction is doubly-digested with both the MSRE and MDRE. When working with relatively pure samples, methylated loci have a Ct from MSRE that is the same as the untreated control, and the Ct obtained from the MDRE is greater. Conversely, unmethylated loci have a MDRE Ct that is identical to untreated and a greater Ct in the MSRE reaction.

The gene *tb2* targeted a 263 bp region from the 5′ end of the gene for the assay. There are four HhaI restriction sites and more than 25 possible mcrBC half-sites (5′-RC-3′) in this region (Figure 4A). We developed a SYBR green real-time PCR assay using the Dynamo Kit from MJ Research (Boston, Massachusetts, United States). The forward primer used was 5′- GCCGCCGCCGACGCCAGCTTTCAC-3′, and the reverse primer was 5′- ATCCCGGGCGCGGTGCATATCTTGCTGTG-3′. The cycling parameters were 95 °C for 3 min, followed by 50 cycles of two-step PCR: 95 °C for 30 s and 70 °C for 30 s. We utilized both a low-temperature (70 °C) and a high-temperature (82 °C) plate read. 2 μg of gDNA was added to a 200-μl reaction cocktail for digestion using the conditions specified by NEB (Beverly, Massachusetts, United States). Half of the sample was digested with 40 U of HhaI overnight, while the other half remained mock-digested. Both “digests” were subsequently split in two, and to each new digestion, cocktails with NEB2, BSA, and 2×GTP were prepared using a final volume of 100 μl. 40 U of mcrBC was added to one of the mock-digested samples and to one of the HhaI-digested samples. All four reactions were incubated overnight at 37 °C. The PCR assays utilized approximately 40 ng from each of the digests. All amplifications were performed in quadruplicate. A standard dilution curve of S. bicolor gDNA in 1× NEB2 was used to ensure linearity of the system. All reactions were verified using melt-curve analysis. Three replicate analyses were performed (digestions and cycling).

Each of the 11 genes was broken into approximately 1.5-kb pieces, which were aligned to create a consensus kafirin assembly (Figure 4B). The consensus kafirin sequence was examined and a 247-bp region was selected. The forward primer was 5′- CTCCTTGCGCTCC TTGCTCTTTC-3′, (where GCGC is a HhaI restriction site) and the reverse primer was 5′- GCTGCGCTGCGATGGTCTGT-3′. We used the same SYBR green real-time PCR assay with the Dynamo Kit (MJ Research), as mentioned above for the *tb2* gene. Cycling parameters were 95 °C for 3 min, followed by 50 cycles of two-step PCR: 95 °C for 30 s and 56 °C for 30 s. We utilized both a low-temperature (70 °C) and a high-temperature (82 °C) plate read. The input of gDNA was cut to 10 ng per reaction. All amplifications were performed in quadruplicate. Three replicate analyses were performed (digestions and cycling). The threshold was set using a template dilution standard control. For the kafirin genes, the average difference in Ct between the mcrBC single and the HhaI + McrBC double-digests is 2.46 cycles (22.08 ± 0.34 *Hha*I + McrBC - 19.62 ± 0.19 McrBC).

PCR products from the kafirin cycling reactions were cloned using the topoisomerase-assisted method (Invitrogen, Carlsbad, California, United States). Libraries of insert-bearing clones were generated using standard techniques. From each library, 200 lacZ-negative clones were selected for characterization. The clones were sequenced with a single read using the M13 priming site on the pCR2.1 plasmid (Invitrogen). All seven subfamilies were discovered from both the treated and untreated genomic samples (unpublished data), indicating that all 11 genes were amplified and recoverable, even in the mcrBC-digested fractions.

#### Identification of DREB1 orthologs in the sorghum dataset

The five *Arabidopsis DREB* genes *DREB1A, DREB1B, DREB1C, DREB2A,* and *DREB2B* were used in a TBLASTN search of an assembly the sorghum dataset using WU-BLAST (parameters: E =e-5; matrix=BLOSUM80; topcomboN=1; wordmask=seg+xnu). Matches to the sorghum assembly with an E-value of 1 × 10^−8^ or less were analyzed with FGENESH (monocot) to select assemblies with a full-length protein. Out of 67 full length proteins identified in this manner, five sorghum proteins were identified as *DREB1* genes based on conservation of the AP2 domain and a conserved C-terminal motif, LWSY [31].

## Supporting Information

Table S1 Arabidopsis Proteins with Homologs in Sorghum but Not Rice Shown is a list of 127 *Arabidopsis* proteins that have matches to the sorghum MF set at a TBLASTN E-value less than or equal to 1 × 10^−8^, but are not found in the *O. s. japonica* or *O. s. indica* genomes at the same cutoff.
(120 KB DOC).

### Accession Numbers

The sorghum MF sequence set is deposited in the Genome Survey Sequence division of GenBank (http://www.ncbi.nlm.nih.gov/). On 6 January 2004, Orion deposited 50,161 of the sequences under accession numbers CL147592–CL197752. The 36,825 Cold Spring Harbor Laboratories MF sequences were previously deposited in GenBank under the accession numbers CC058553–CC059980, BZ329127–BZ342789, BZ342901–BZ352342, BZ365856–BZ368372, BZ369686–BZ370012, BZ421595–BZ424357, BZ625682–BZ629992, and BZ779555–BZ78192. The remaining 481,989 MF sequences from Orion Genomics are deposited in GenBank under accession numbers CL147592–CL197752 and CW020594–CW502582. The Orion UF sequences are deposited in GenBank's Genome Survey Sequence under accession numbers CW512190–CW514008. The University of Oklahoma UF sequences are deposited in the NCBI trace archive under accession numbers TI566112507–TI566128395. GenBank accession numbers for other genes discussed in this paper are sorghum *Cs1* (AF206660); *Arabidopsis DREB* genes *DREB1A* (Q9M0L0), *DREB1B* (P93835), *DREB1C* (Q9SYS6), *DREB2A* (O82132), and *DREB2B* (O82133). Genbank accession numbers for BAC clones (with GenInfo identifiers) are AC120496.1 (GI:20486389), AF010283.1 (GI:2735839), AF061282.1 (GI:4539654), AF114171.1 (GI:4680196), AF124045.1 (GI:5410347), AF369906.1 (GI:19851516), AF466199.1 (GI:18390096), AF466200.1 (GI:18481699), AF466201.1 (GI:18483227), AF466204.1 (GI:18568251), AF503433.1 (GI:21326110), AF527807.1 (GI:22208458), AF527808.1 (GI:22208471), and AF527809.1 (GI:22208503). The GenBank accession number for the protein CAPRICE is NP_182164. Accession numbers for genes used in phylogenetic analysis of sorghum *DREB* are as follows. Rice genes are *OsDREB1A* (AF300970), *OsDREB1B* (AF300972), *OsDREB1C* (AP001168, nucleotides 142337–142981), and *OsDREB1D* (AB023482, nucleotides1489–2250); AP2 domains from other *Arabidopsis* proteins are also included: *APETALA2* (R2 domain, accession number P47927), *AtERF-1* (BAA32418), *LEAFY PETIOLE* (AAF32292), and *TINY* (Q39127).

## Abbreviations

5 mC: 5′ methylated cytosine
BAC: bacterial artificial chromosome
bp: basepairs
CDS: coding sequence
Ct: cycle threshold
DREB: dehydration-responsive element binding protein
EST: expressed sequence tag
E-value: BLAST Expect value
FP: filter power
gDNA: genomic DNA
HC: high C_0_ *t*
kb: kilobase
Mb: megabase
mcrBC: modified cytosine restriction
MDRE: methylation-dependent restriction enzyme
MF: methylation filtration/filtering/filtered
miRNA: microRNA
MSRE: methylation-sensitive restriction enzyme
TNR: trinucleotide repeat
UF: unfiltered
