# Supplementary material for: Sorghum Genome Sequencing by Methylation Filtration
Source: PLoS Biol. 2005 Jan 4;3(1):e13. doi: 10.1371/journal.pbio.0030013 (PMC539327; doi:10.1371/journal.pbio.0030013)
Supplement: Table S1 — Shown is a list of 127 Arabidopsis proteins that have matches to the sorghum MF set at a TBLASTN E-value less than or equal to 1 × 10^−8^, but are not found in the O. s. japonica or O. s. indica genomes at the same cutoff. (120 KB DOC). [file pbio.0030013.st001.doc]

| **Accession** | **Definition** |
| --- | --- |
| NP_172574.1 | proline-rich protein family [Arabidopsis thaliana] |
| NP_172845.1 | hypothetical protein [Arabidopsis thaliana] |
| NP_172920.1 | hypothetical protein [Arabidopsis thaliana] |
| NP_173381.1 | bZIP protein [Arabidopsis thaliana] |
| NP_173644.2 | expressed protein [Arabidopsis thaliana] |
| NP_173939.1 | hypothetical protein [Arabidopsis thaliana] |
| NP_174482.1 | F-box protein family [Arabidopsis thaliana] |
| NP_174864.1 | hypothetical protein [Arabidopsis thaliana] |
| NP_174888.1 | zinc finger protein -related [Arabidopsis thaliana] |
| NP_175070.1 | CHP-rich zinc finger protein, putative [Arabidopsis thaliana] |
| NP_175177.1 | hypothetical protein [Arabidopsis thaliana] |
| NP_175283.1 | hypothetical protein [Arabidopsis thaliana] |
| NP_175741.1 | CHP-rich zinc finger protein, putative [Arabidopsis thaliana] |
| NP_175890.1 | hypothetical protein [Arabidopsis thaliana] |
| NP_176689.1 | hypothetical protein [Arabidopsis thaliana] |
| NP_177044.1 | MutT/nudix family protein [Arabidopsis thaliana] |
| NP_178180.1 | expressed protein [Arabidopsis thaliana] |
| NP_178366.1 | CHP-rich zinc finger protein, putative [Arabidopsis thaliana] |
| NP_178545.1 | CHP-rich zinc finger protein, putative [Arabidopsis thaliana] |
| NP_178612.1 | glycine-rich protein [Arabidopsis thaliana] |
| NP_178619.1 | glycine-rich protein [Arabidopsis thaliana] |
| NP_179008.1 | CHP-rich zinc finger protein, putative [Arabidopsis thaliana] |
| NP_179237.1 | F-box protein family [Arabidopsis thaliana] |
| NP_179598.1 | hypothetical protein [Arabidopsis thaliana] |
| NP_179892.1 | late embryogenesis abundant proteins -related [Arabidopsis thaliana] |
| NP_180653.1 | expressed protein [Arabidopsis thaliana] |
| NP_180925.1 | expressed protein [Arabidopsis thaliana] |
| NP_181485.2 | expressed protein [Arabidopsis thaliana] |
| NP_181534.1 | CHP-rich zinc finger protein, putative [Arabidopsis thaliana] |
| NP_181851.1 | CHP-rich zinc finger protein, putative [Arabidopsis thaliana] |
| NP_181967.1 | CHP-rich zinc finger protein, putative [Arabidopsis thaliana] |
| NP_182164.1 | myb-related protein CAPRICE (CPC) [Arabidopsis thaliana] |
| NP_182220.1 | hypothetical protein [Arabidopsis thaliana] |
| NP_187273.1 | transcriptional factor B3 family [Arabidopsis thaliana] |
| NP_187356.1 | CHP-rich zinc finger protein, putative [Arabidopsis thaliana] |
| NP_188085.1 | hypothetical protein [Arabidopsis thaliana] |
| NP_188089.1 | F-box protein family [Arabidopsis thaliana] |
| NP_188168.1 | hypothetical protein [Arabidopsis thaliana] |
| NP_188786.1 | copper-binding protein family [Arabidopsis thaliana] |
| NP_188978.1 | protein phosphatase 2C (PP2C), putative [Arabidopsis thaliana] |
| NP_189200.1 | hypothetical protein [Arabidopsis thaliana] |
| NP_189256.1 | CHP-rich zinc finger protein, putative [Arabidopsis thaliana] |
| NP_189287.1 | CHP-rich zinc finger protein, putative [Arabidopsis thaliana] |
| NP_189308.1 | light regulated protein -related [Arabidopsis thaliana] |
| NP_189384.1 | CHP-rich zinc finger protein, putative [Arabidopsis thaliana] |
| NP_189385.1 | CHP-rich zinc finger protein, putative [Arabidopsis thaliana] |
| NP_189386.1 | CHP-rich zinc finger protein, putative [Arabidopsis thaliana] |
| NP_189537.1 | AIG2-related protein [Arabidopsis thaliana] |
| NP_190132.1 | hypothetical protein [Arabidopsis thaliana] |
| NP_190299.2 | F-box protein family [Arabidopsis thaliana] |
| NP_190873.1 | expressed protein [Arabidopsis thaliana] |
| NP_190972.1 | photoassimilate-responsive protein PAR-1b -related protein [Arabidopsis thaliana] |
| NP_191492.1 | hypothetical protein [Arabidopsis thaliana] |
| NP_191791.1 | hypothetical protein [Arabidopsis thaliana] |
| NP_192164.1 | expressed protein [Arabidopsis thaliana] |
| NP_192444.1 | zinc finger (C3HC4-type RING finger) protein family [Arabidopsis thaliana] |
| NP_192715.2 | expressed protein [Arabidopsis thaliana] |
| NP_192767.1 | hypothetical protein [Arabidopsis thaliana] |
| NP_192958.1 | zinc finger (C3HC4-type RING finger) protein family [Arabidopsis thaliana] |
| NP_192973.1 | protease inhibitor/seed storage/lipid transfer protein (LTP) family [Arabidopsis thaliana] |
| NP_193039.2 | expressed protein [Arabidopsis thaliana] |
| NP_193520.1 | hypothetical protein [Arabidopsis thaliana] |
| NP_193829.2 | bHLH protein family [Arabidopsis thaliana] |
| NP_194201.1 | expressed protein [Arabidopsis thaliana] |
| NP_194288.2 | expressed protein [Arabidopsis thaliana] |
| NP_194795.1 | stress responsive protein homolog [Arabidopsis thaliana] |
| NP_194859.1 | AIG2-related protein [Arabidopsis thaliana] |
| NP_194930.1 | expressed protein [Arabidopsis thaliana] |
| NP_195166.1 | transcriptional factor B3 family [Arabidopsis thaliana] |
| NP_195390.1 | hypothetical protein [Arabidopsis thaliana] |
| NP_195853.1 | CHP-rich zinc finger protein, putative [Arabidopsis thaliana] |
| NP_195854.1 | CHP-rich zinc finger protein, putative [Arabidopsis thaliana] |
| NP_196084.1 | MADS-box protein [Arabidopsis thaliana] |
| NP_196131.1 | zinc finger (C2H2 type) protein family [Arabidopsis thaliana] |
| NP_196161.1 | immunophilin / FKBP-type peptidyl-prolyl cis-trans isomerase, putative [Arabidopsis thaliana] |
| NP_196220.1 | hypothetical protein [Arabidopsis thaliana] |
| NP_196896.1 | hypothetical protein [Arabidopsis thaliana] |
| NP_197301.1 | transcriptional factor B3 family [Arabidopsis thaliana] |
| NP_197435.2 | expressed protein [Arabidopsis thaliana] |
| NP_197885.1 | hypothetical protein [Arabidopsis thaliana] |
| NP_198140.1 | hypothetical protein [Arabidopsis thaliana] |
| NP_198218.1 | hypothetical protein [Arabidopsis thaliana] |
| NP_198414.1 | hypothetical protein [Arabidopsis thaliana] |
| NP_198544.1 | zinc finger (C3HC4-type RING finger) protein family [Arabidopsis thaliana] |
| NP_198606.1 | seven in absentia (sina) protein family [Arabidopsis thaliana] |
| NP_198925.1 | No apical meristem (NAM) protein family [Arabidopsis thaliana] |
| NP_199098.1 | CHP-rich zinc finger protein, putative [Arabidopsis thaliana] |
| NP_199118.1 | CHP-rich zinc finger protein, putative [Arabidopsis thaliana] |
| NP_199178.1 | bHLH protein family [Arabidopsis thaliana] |
| NP_199585.1 | expressed protein [Arabidopsis thaliana] |
| NP_199756.1 | bZIP family transcription factor [Arabidopsis thaliana] |
| NP_200052.1 | photoassimilate-responsive protein PAR-related protein [Arabidopsis thaliana] |
| NP_200389.1 | CHP-rich zinc finger protein, putative [Arabidopsis thaliana] |
| NP_200391.1 | CHP-rich zinc finger protein, putative [Arabidopsis thaliana] |
| NP_200466.1 | expressed protein [Arabidopsis thaliana] |
| NP_200935.1 | bHLH protein family [Arabidopsis thaliana] |
| NP_201179.1 | hypothetical protein [Arabidopsis thaliana] |
| NP_201239.1 | expressed protein [Arabidopsis thaliana] |
| NP_201556.1 | hypothetical protein [Arabidopsis thaliana] |
| NP_563619.1 | transcription co-activator (SYT2) -related [Arabidopsis thaliana] |
| NP_564659.1 | expressed protein [Arabidopsis thaliana] |
| NP_564695.1 | awaiting functional assignment [Arabidopsis thaliana] |
| NP_564776.1 | expressed protein [Arabidopsis thaliana] |
| NP_565043.1 | expressed protein [Arabidopsis thaliana] |
| NP_565661.2 | hypothetical protein [Arabidopsis thaliana] |
| NP_565860.1 | chloroplast lumen common protein family [Arabidopsis thaliana] |
| NP_565907.1 | expressed protein [Arabidopsis thaliana] |
| NP_565993.1 | expressed protein [Arabidopsis thaliana] |
| NP_566146.1 | arabinogalactan-protein (AGP11) [Arabidopsis thaliana] |
| NP_567100.1 | expressed protein [Arabidopsis thaliana] |
| NP_567194.1 | SSXT protein family [Arabidopsis thaliana] |
| NP_567222.1 | zinc finger (C3HC4-type RING finger) protein family [Arabidopsis thaliana] |
| NP_567381.1 | expressed protein [Arabidopsis thaliana] |
| NP_567516.1 | expressed protein [Arabidopsis thaliana] |
| NP_567592.1 | GCN5-related N-acetyltransferase (GNAT) family [Arabidopsis thaliana] |
| NP_567750.1 | peptidylprolyl isomerase, putative [Arabidopsis thaliana] |
| NP_567899.1 | ferredoxin - related [Arabidopsis thaliana] |
| NP_671841.1 | hypothetical protein [Arabidopsis thaliana] |
| NP_680182.1 | expressed protein [Arabidopsis thaliana] |
| NP_680707.1 | GATA zinc finger protein -related [Arabidopsis thaliana] |
| NP_683415.1 | transcriptional factor B3 family [Arabidopsis thaliana] |
| NP_849289.1 | expressed protein [Arabidopsis thaliana] |
| NP_849371.1 | expressed protein [Arabidopsis thaliana] |
| NP_849937.1 | glycine-rich protein [Arabidopsis thaliana] |
| NP_850389.1 | expressed protein [Arabidopsis thaliana] |
| NP_850392.1 | hypothetical protein [Arabidopsis thaliana] |
| NP_850876.1 | heavy-metal-associated domain-containing protein [Arabidopsis thaliana] |
